# Predictors for achieving adequate protein and energy intake in nursing home rehabilitation patients

**DOI:** 10.1007/s40520-017-0850-4

**Published:** 2017-11-17

**Authors:** J. I. van Zwienen-Pot, M. Visser, H. M. Kruizenga

**Affiliations:** 1 Dutch Malnutrition Steering Group, Nicolaas Witsenkade 13hs, 1017 ZR Amsterdam, The Netherlands; 2 Department of Dietetics, Zorgpartners Midden-Holland, Ronsseweg 410, 2803 ZX Gouda, The Netherlands; 3 Department of Health Sciences, Faculty of Earth and Life Sciences, VU University, De Boelelaan 1085, 1081 HV Amsterdam, The Netherlands; 4 Department of Nutrition and Dietetics, Internal Medicine, VU University Medical Center, PO Box 7057, 1007 MB Amsterdam, The Netherlands

**Keywords:** Older adults, Nursing home rehabilitation, Adequate protein and energy intake, Predictors, Undernutrition, Dietetic treatment

## Abstract

**Background:**

Adequate energy and protein intake could be essential for contributing significantly to the rehabilitations process. Data on the actual nutritional intake of older nursing home rehabilitation patients have not yet been investigated.

**Aims:**

To investigate the nutritional intake and predictors for achieving protein and energy requirements on the 14th day of admission in nursing home rehabilitation patients.

**Methods:**

Fifty-nine patients aged 65+ years newly admitted to nursing home rehabilitation wards were included. Data on potential variables were collected on admission. On the fourteenth day nutritional intake was assessed. Intake was considered ‘adequate’ if patients had achieved ≥ 1.2 g of protein/kg bodyweight and ≥ 85% of their energy needs according to Harris and Benedict + 30%. Multiple logistic regression analyses were performed to select predictors for adequate intake.

**Results:**

Protein and energy intake was assessed in 79 patients [67% female, mean age 82 ± (SD) 8 years, BMI 25 ± 6 kg/m^2^]. Mean energy intake was 1677 kcal (± 433) and mean protein intake was 68 g (± 20). Fourteen patients (18%) achieved an adequate protein and energy intake. Predictors for adequate intake were use of sip/tube feeding (OR = 7.7; 95% CI = 1.35–44.21), BMI (0.68; 0.53–0.87) and nausea (8.59; 1.42–52.01).

**Conclusion:**

Only 18% of older nursing home rehabilitation patients had an adequate protein and energy intake at 14 days after admission. Patients with higher BMI were less likely, while those using sip/tube feeding or feeling nauseous were more likely to achieve an adequate protein and energy intake.

## Introduction

As a result of the phenomenon ‘population ageing’, the number of older adults admitted to rehabilitation wards of nursing homes has increased [1]. Rehabilitation in nursing homes is required after an accident, surgery or hospitalization when immediate return to the usual living situation is not possible. Rehabilitation in Dutch nursing homes consists of integrated multidisciplinary care for frail older adults, focusing on expected recovery of function and participation [2].

The recovery from illness or injury often requires patients to undergo a period of muscle disuse. Muscle disuse for only 4 days already causes a decline in functional strength in older adults [3]. Muscle strength is a critical component in maintaining and improving physical function, mobility, and vitality in older adults [4]. Thus, for the purpose of nursing home rehabilitation it is essential to attenuate muscle loss during a period of limited muscle use and, where possible, allow patients to increase in muscle strength.

Both nutrition and muscle contraction are anabolic stimuli and therefore, necessary to reduce the loss of muscle mass and strength. Protein intake has been shown to stimulate skeletal muscle protein synthesis and prevents protein degradation, resulting in a positive protein balance [5–9]. An imbalance between protein intake and protein requirement can result in loss of skeletal muscle mass [10].

Older adults with weight loss lose significantly more lean mass and strength than those who either maintain or gain weight [4].

Consequently, an adequate dietary intake that meets the energy and protein requirements could be essential to optimize muscle mass and strength, contributing significantly to the rehabilitations process.

Unfortunately, data on the actual nutritional intake of older rehabilitation patients is scarce, especially for rehabilitation wards of nursing homes. According to studies in (older) hospitalized patients, 21–74% of all patients had an inadequate protein and energy intake [11–13], independent of their nutritional status. Whether this percentage also applies to older adult rehabilitation patients in nursing homes has not yet been investigated. Furthermore, it is unclear which subgroups of patients are more likely to have an adequate energy-protein intake.

The objectives of this study were to investigate the nutritional intake of Dutch nursing home rehabilitation patients on the 14th day following admission and to explore the predictors of an adequate energy-protein intake on this day.

## Materials and methods

### Subjects

This study was carried out in three nursing home rehabilitation wards of *Zorgpartners Midden-Holland*, located in the Netherlands. These rehabilitation wards provide temporary care for older patients recovering from their illness or injuries. From March 2013 until February 2014, patients 65 years-of-age or older admitted to these rehabilitation wards were screened within the first week of admission to determine eligibility based on current medical records. All participants received written and verbal information regarding the purpose of the study prior to signing an informed consent form. Patients with severe cognitive impairment (as judged by health care providers or family), non-Dutch/English speaking patients and patients with an expected length of stay of less than 2 weeks were excluded.

A convenience sample was recruited by identifying all patients admitted to the wards within the preceding 24 h at the time that the researcher was available (usually 2 days a week).

The research protocol was approved by the Ethics Review board of the VU University Medical Center and the ethics committee of *Zorgpartners Midden-Holland*.

### Outcome measurement: adequate protein and energy intake

On the 14th day following admission the actual protein and energy intake was assessed using a face-to-face 24-h recall method. This time period was selected to exclude the first days of rehabilitation where patients may not yet be accustomed to their new living situation and dietary care. The 24-h recall method gives a detailed description of all foods and beverages consumed, including oral nutritional support or tube feeding, during the previous 24-h period. Protein and energy intake was calculated, respectively, in g and kcal, based on the NEVO Dutch Food Consumption Table 2006 [14].

Adequate protein intake was defined as 1.2 g/kg bodyweight per day [15, 16]. For obese patients, weight was adjusted to a BMI of 27.5 kg/m^2^ [17]. Adequate energy intake was based on the estimated resting energy expenditure of Harris and Benedict [18] plus an additional factor of 30% for activity and/or disease-related energy expenditure [19]. Because this requirement is an estimation, a cut off value of 85% was used to define an adequate energy intake.

### Potential predictors of adequate protein and energy intake

To identify predictors of adequate protein and energy intake, social, medical, functional, psychological and nutritional status variables were measured in the first week of admission. On the 14th day following admission current dietetic treatment variables were recorded.

#### Level of education

Patients were asked to indicate the highest level of education completed: (1) Lower general education, (2) Lower vocational education, (3) Intermediate general education, (4) Intermediate vocational education, (5) Secondary general education, (6) Higher vocational education, (7) Scientific education. Education level was categorized into low (1, 2), medium (3–5) and high (6,7).

#### Medical variables

Of each patient a complete list of all primary (reason for admission) and secondary diagnoses (co morbidity) was obtained from the current medical records and hospital discharge records. Patients were categorized according to their reason for admission: (1) Trauma, (2) Elective orthopaedics, (3) Cerebrovascular accident (CVA) and (4) Other. Trauma included patients with an operative hip fracture, vertebral fractures, fractures of the femur, humerus fractures, pelvic fractures or other fractures. Elective orthopaedics included patients with a joint replacement, generally a hip or knee, or a revision of a previously placed prosthesis. The category CVA consisted of patients affected by a stroke or brain haemorrhage. The category Other contained a variety of diseases, but mostly diseases of the cardiovascular system or lungs, and disorders of the musculoskeletal system.

Nausea was evaluated by a patient’s subjective sensation of feeling nauseous, and answering categories with yes and no. Appetite during the last week was assessed by asking ‘Have you experienced a decreased appetite in the past week?’ (yes/no).

Problems with chewing and swallowing were assessed by asking ‘Do you experience difficulties with chewing or swallowing?’ (yes/no).

Pain was assessed using a subscale of the Nottingham Health Profile [20] The pain questionnaire included the following five items: (1) ‘I am in pain when I am standing,’ (2) ‘I find it painful to change position,’ (3) ‘I am in pain when I am sitting,’ (4) ‘I am in pain when I walk,’ (5) ‘I am in constant pain,’ with response categories yes and no. Sum scores were calculated ranging from no symptoms of pain—five symptoms of pain.

### Nutritional status variables

Before anthropometric measurements were taken, patients were asked two questions about the perception of their nutritional status and current weight: (1) ‘What do you think of the weight that you have right now?’ with response options: severely underweight, underweight, normal weight, overweight, severely overweight, and (2) ‘Do you currently find yourself undernourished?’ (yes/no).

During the first week of admission patients were weighed on a calibrated weighing chair or platform. Weight was recorded to the nearest of 0.1 kg. Knee height was measured using the distance from the sole of the foot to the anterior surface of the thigh with ankle and knee each flexed to a 90° angle and a flexible, non-stretchable measuring tape (Seca) and was recorded to the nearest 0.5 cm. With a formula developed in Dutch older persons [21] the total body height was estimated from knee height. When it was impossible to measure knee height because of special shoes that could not be removed, or presence of bandages, the height from an identity card or a passport was used (*N* = 9). BMI (kg/m^2^) was calculated by dividing the weight (kg) by height squared (m).

Weight loss during the past month and past 6 months were obtained by verbally asking the patient or using body weights documented in the patient records (*n* = 2).

Nutritional status was categorized as: severely undernourished (> 10% unintentional weight loss in the past 6 months and/or > 5% unintentional weight loss in the past month and/or BMI ≤ 20 kg/m^2^), moderately undernourished (5–10% unintentional weight loss in the past 6 months and/or BMI 20.01–22 kg/m^2^), well-nourished (< 5% unintentional weight loss in the past 6 months, BMI 22.01–28 kg/m^2^) and overweight (BMI > 28 kg/m^2^) [22–24].

Mid upper arm circumference (MUAC) of the non-dominant arm midway between the bony protrusion on the shoulder (acromion) and the point of the elbow (olecranon) was measured in duplicate to the nearest 0.5 cm, using a flexible, non-stretchable measuring tape (Seca). The mean value of the two measurements was used in the analysis.

Bioelectrical impedance analysis (BIA) was used to estimate the fat free mass. Body resistance (R, Ohm) was measured at a frequency of 50 kHz using a Bodystat 1500 MDD (Euromedix, Belgium). Fat free mass (kg) was predicted with the equation of Kyle et al. [25]. Fat free mass index (FFMI) was calculated as fat free mass divided by height squared. An FFMI below the tenth percentile of the reference values of Schutz et al. were categorised as low [26]. FFMI was used in the analysis as a continuous variable.

#### Functional variables

Functional ability was assessed with the Barthel index [27] and Functional Ambulant Classification (FAC) [28]. The Barthel index provides an indication of a patient’s performance on ten activities of daily living (ADL) functions: bowel and bladder care, grooming, toilet use, transfers, ambulation, dressing, stair climbing and bathing. The total score ranged from 0 to 20; 0–4 points: Total dependence, 5–9 points: Severe dependence, 10–14 points: Moderate dependence, 15–19 points: Slight dependence, 20 points: ADL independence. Ambulatory ability was defined using FAC as one of six functional levels of ambulation: FAC 0: Non functional Ambulation, FAC 1: Ambulator-Dependent for physical assistance Level II, FAC 2: Ambulatory-Dependent for physical assistance Level I, FAC 3: Ambulator-Dependent for supervision, FAC 4: ambulator-Independent level surfaces only and FAC 5: Ambulator-Independent.

Need for assistance when eating was assessed by using the ‘feeding item’ of the Barthel index [27], with answering categories: (0) unable, (1) needs help cutting, spreading butter (2) independent (food provided within reach).

Handgrip strength (kg) was measured using a calibrated hydraulic hand dynamometer (Saehan, Masan corporation, Korea). Each patient performed two maximum grip strength trials with each hand, while seated with the shoulders adducted, elbows flexed 90°, and the forearms in neutral position according to the American Society of Hand Therapists recommendations [29] Both maximal values are recorded to the nearest 0.5 kg, and the mean of the two measurements was used. A handgrip strength below the tenth percentile of the reference values of Bohannon et al. was categorised as low [30].

#### Psychological variable

The subscale Mental Component Summary (MCS) derived from the SF-12 was used to assess mental health. The SF-12 questionnaire comprised 12 items (questions) and 8 scales including physical functioning, role limitations due to physical health, general health, bodily pain, social functioning, vitality, mental health and role limitation due to emotional health. MCS scores were computed using the scores of the twelve questions and ranged from 0 (lowest level of psychological health) to 100 [31].

#### Dietetic treatment variables

On day 14, current information regarding dietetic treatment received following admission was retrieved from patient records. Furthermore, current data on the use of sip feeding or tube feeding (yes/no) received following admission were recorded.

### Statistics

Descriptive statistics were used to express means, standard deviations, percentages and frequencies to describe patients’ characteristics according to adequacy of protein and energy intake on day 14 following admission (yes/no).

Univariate associations between all potential predictors and adequate intake were analysed using logistic regression analysis. A prediction model was made with adequate protein and energy intake as the dependent, dichotomous variable, using multiple logistic regression analysis. Preliminary assumption testing was conducted to ensure no violation of the assumptions, including multicollinearity. Multicollinearity was assessed using the variance inflation factors (VIF) and tolerance values. In the case of a VIF value above 5, one of the involved determinants was removed from the multiple model due to collinearity. The study sample was relatively small, hence only a limited number of variables could be included in the regression analyses. Only the significant variables according to the univariate analyses were included in the multiple model using the backward selection method with a significance level of *P* ≤ 0.10. Furthermore, the explained variance of the prediction model was determined with Nagelkerke’s R2, reflecting the proportion of variation in the outcome explained by the predictors in the model.

Sensitivity analyses were performed in which the patients discharged to home or self-care were part of the adequate nutrition category. The rationale was that these individuals might have a better chance of achieving adequate protein and energy intake. Furthermore, univariate associations between all potential predictors and an intake of 0.8 g/kg bodyweight (the current recommended daily intake of protein for older persons [32, 33]) were analysed using logistic regression analysis. Another prediction model was made with the current guideline as the dependent, dichotomous variable, using multiple logistic regression analysis.

Results are presented as odds ratios (OR) with 95% confidence intervals (CIs). A *P* value of < 0.05 was considered as statistically significant for descriptive statistics and univariate analyses. All analyses were performed using IBM spss statistics 22.0.

## Results

### Patient characteristics

**Fig. 1:**
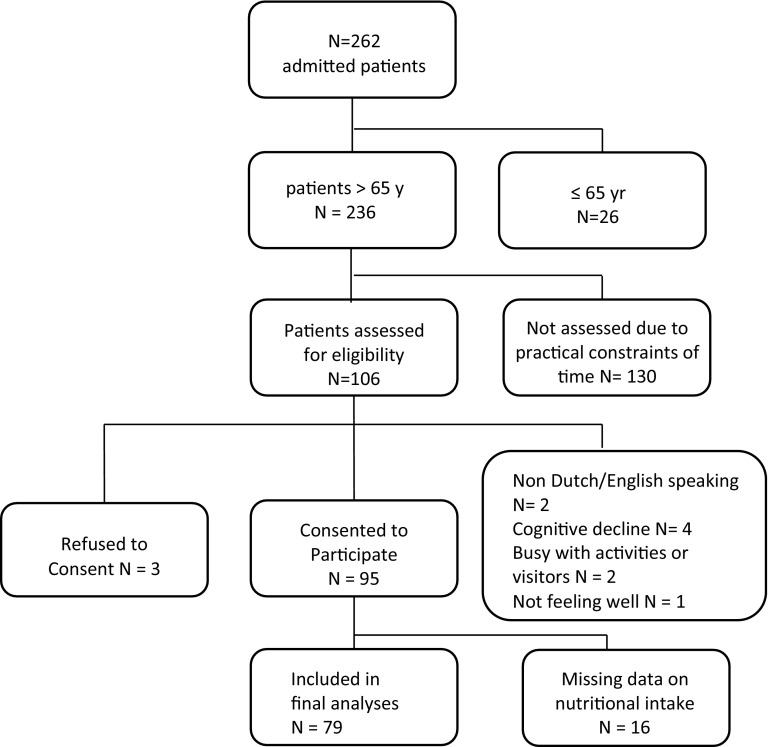
Flow chart

During the study period, 262 patients were admitted for rehabilitation of whom 26 were younger than 65 years and 130 were admitted on days when the researcher was not available the following day. Altogether, 106 patients were further assessed for eligibility of whom 3 declined participation and 9 were not eligible for enrolment (Fig. 1).

A total of 95 patients were included in the study. Sixteen patients (17%) did not provide adequate data on energy and protein intake on the 14th day following admission due to various reasons: discharged to home or self-care (*n* = 11), hospital admission (*n* = 2), deceased (*n* = 1), no longer willing to participate (*n* = 1), or transferred to another rehabilitation facility (*n* = 1).

Mean age of the 79 patients with complete data was 81.8 (± 7.6) years and 53 patients were female (67%) (Table 1). Admission reasons were trauma (40%), Elective orthopaedics (15%), CVA (12%) and Other (33%). The category ‘Other’ contained mostly disorders of the musculoskeletal system (24%), respiratory system (14%) and the cardiovascular system (14%).

**Table 1:** Characteristics of older rehabilitation patients included in the study, also stratified by adequate and inadequate protein and energy intake as assessed at 14 days following admission

Measured in the first week following admission	All	Energy and protein intake on day 14	
		Adequate	Inadequate
N patients	79	14 (17.7)	65 (82.3)
Energy intake (% of requirements Mean ± SD)	95.2 ± 27.9	134.8 ± 24.5	87.5 ± 21.3
Protein intake (% of requirements Mean ± SD)	79.1 ± 29.2	126.7 ± 29.9	69.8 ± 17.9
Age (year, Mean ± SD)	81.8 ± 7.6	82.6 ± 8.2	81.7 ± 7.5
Female *n* (%)	53 (67.1)	9 (64.3)	44 (67.7)
Level of education			
Education ^b^: low *n* (%)	51 (65.4)	10 (71.4)	41 (64.1)
Medium *n* (%)	24 (30.8)	3 (21.4)	21 (32.8)
High *n* (%)	3 (3.8)	1 (7.1)	2 (3.1)
Medical variables			
Trauma *n* (%)	32 (40.5)	6 (42.9)	26 (40.0)
Elective orthopaedics *n* (%)	12 (15.2)	–	12 (18.5)
CVA *n* (%)	9 (11.9)	1 (7.1)	8 (12.3)
Other *n* (%)	26 (32.9)	7 (50.0)	19 (29.2)
Nausea *n* (%)	16 (20.2)	6 (42.9)	10 (15.4)
Loss of appetite *n* (%)	46 (58.2)	11 (78.6)	35 (53.8)
Subjective pain Mean ± SD	2.4 (1.8)	2.4 (2.0)	2.4 (1.8)
Nutritional status variables			
BMI kg/m^2^ Mean ± SD	25.3 ± 5.8	21.0 ± 3.1	26.2 ± 5.9
Patients’ perception on weight status			
Underweight *n* (%)	18 (22.8)	8 (57.1)	10 (15.4)
Normal weight *n* (%)	46 (58.2)	6 (42.9)	40 (61.5)
Overweight *n* (%)	15 (19.0)	–	15 (23.1)
Self-perceived undernutrition	8 (10.1)	6 (42.9)	2 (3.1)
Percentage weight loss 6 months Mean ± SD	4.1 ± 5.6	9.1 ± 7.6	3.0 ± 4.5
FFMI kg/m^2^ mean ± SD ^c^	17.0 ± 2.8	15.3 ± 1.8	17.4 ± 2.8
Undernutrition: well-nourished *n* (%)	44 (55.7)	3 (21.4)	41 (63.1)
Moderate undernourished *n* (%)	13 (16.5)	3 (21.4)	10 (15.4)
Severely undernourished *n* (%)	22 (27.8)	8 (57.1)	14 (21.5)
Functional variables			
Barthel score Mean ± SD	10.8 ± 3.9	11.6 ± 4.1	10.6 ± 3.9
FAC 0	11 (13.9)	1 (7.1)	10 (15.4)
FAC 1	7 (8.9)	–	7 (10.8)
FAC 2	19 (24.1)	2 (14.3)	17 (26.2)
FAC 3	25 (31.6)	7 (50.0)	18 (27.7)
FAC 4	17 (21.5)	4 (28.6)	13 (20.0)
FAC 5	–	–	–
Feeding item: unable *n* (%)	2 (2.5)	1 (7.1)	1 (1.5)
Feeding item: needs help *n* (%)	22 (27.8)	3 (21.4)	19 (29.2)
Feeding item : independent *n* (%)	55 (69.6)	10 (71.4)	45 (69.2)
Difficulties with chewing or swallowing *n* (%)	13 (16.5)	5 (35.7)	8 (12.3)
Handgrip strength kg, Mean ± SD ^e^	17.9 ± 7.7	18.4 ± 8.8	17.8 ± 7.5
Psychological variable			
SF12 poor mental quality of life, MCS score < 50 n (%) ^f^	56 (73.7)	11 (78.5)	45 (72.5
Measured on day 14			
Dietetic treatment variables			
Dietetic treatment *n* (%)	40 (50.6)	11 (78.6)	29 (44.6)
Sip/tube feeding *n* (%)	12 (15.2)	6 (42.9)	6 (9.2)

Adequate: patients who achieved an intake of ≥ 85% of REE of Harris and Benedict plus an additional factor of 30% and an intake of ≥ 1.2 g of protein/kg bodyweight per day (bodyweight was adjusted to a BMI of 27.5 for obese patients)

*FFMI* fat free mass index, *FAC* functional ambulation categories, *MCS score* mental component summary

^b^Data missing for 1 patient

^c^Data missing for 16 patients

^d^Data missing for 2 patients

^e^Data missing for 5 patients

^f^Data missing for 3 patients

A total of 22 patients (28%) were severely undernourished and 13 patients (17%) were moderately undernourished. Mean BMI was 25.3 kg/m^2^ (± 5.8). Based on BIA data from 63 patients, 15 patients (24%) had a low FFMI. A low handgrip strength was present in 36 patients (49% of 74 patients). In forty patients (50%) a dietitian was involved in the treatment.

### Protein and energy intake

**Fig. 2:**
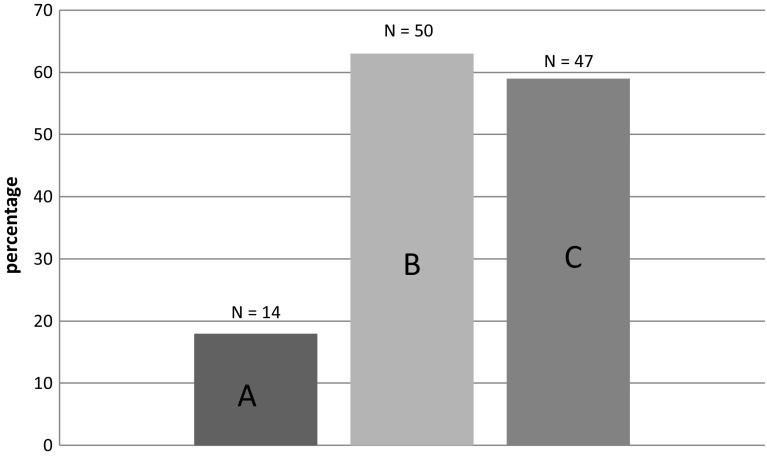
Protein and energy intake of older nursing home rehabilitation patients on the 14th day following admission. A Adequate protein and energy defined as: ≥ 1.2 g of protein/kg bodyweight per day (bodyweight was adjusted to a BMI of 27.5 for obese patients) and ≥ 85% of REE of Harris and Benedict + additional factor of 30%. B Adequate energy defined as: ≥ 85% of REE of Harris and Benedict + additional factor of 30%. C Adequate protein (per current guideline) and energy: ≥ 0.8 g of protein/kg bodyweight per day (bodyweight was adjusted to a BMI of 27.5 for obese patients) and ≥ 85% of REE of Harris and Benedict + additional factor of 30%

Mean intake of all patients was 68 (± 20) grams of protein and 1677 (± 433) kcal. Fourteen patients (18%) met both adequate protein and energy intake (defined as ≥ 85% of REE of Harris and Benedict + additional factor of 30% and ≥ 1.2 grams of protein/kg bodyweight per day). None of the patients had solely adequate protein intake and 36 patients (46%) had an adequate energy intake only. A total of 47 patients (59%) achieved an intake of ≥ 0.8 g of protein/kg bodyweight per day (current guideline) and ≥ 85% of their estimated energy needs (Fig. 2).

Mean intake of patients with an adequate protein and energy intake was 90 (± 24) g of protein and 2103 (± 501) kcal. These patients achieved on average 127(± 30) percent of their protein needs and 135 (± 25) percent of their energy needs. Mean intake of the 14 patients with an inadequate intake was 63 (± 15) g of protein and 1585 (± 360) kcal. This represents an average of 70 (± 18) percent of protein needs and 88 (± 21) percent of energy needs.

### Predictors for adequate protein and energy intake

The results of the univariate associations between potential predictors and adequate protein and energy intake are shown in Table 2. Nausea, receiving dietetic treatment, sip or tube feeding, self-perceived undernutrition, higher percentage of weight loss in the past 6 months, undernutrition and reported difficulties with chewing and swallowing were statistically significantly associated with adequate protein and energy intake on day 14 after admission.

**Table 2:** Univariate associations between patient characteristics and protein and energy intake 14 days following admission in older rehabilitation patients

Measured in the first week following admission	Adequate protein and energy intake^a^				Sensitivity analysis: 0.8 g kg/bw (current guideline)^b^		
	OR	95% CI	*P*		OR	95% CI	*P*
Age (year)	1.02	0.94–1.10	0.65		1.03	0.97–1.09	0.36
Female	0.86	0.27–2.90	0.81		0.69	0.26–1.83	0.46
Level of education							
Education^c^: low (reference)	1.00	–	–		1.00	–	–
Medium	0.59	0.15–2.36	0.45		1.26	0.47–3.42	0.64
High	2.05	0.17–24.92	0.57		5.34	0.26–108.70	0.28
Medical variables							
Trauma	1.13	0.35–3.62	0.84		1.24	0.49–3.10	0.65
Elective orthopaedics	0.15	0.01–2.64	0.19		0.28	0.08–1.02	0.05
CVA	0.55	0.06–4.77	0.59		6.36	0.75–53.60	0.09
Other	2.42	0.75–7.85	0.14		0.90	0.35–2.32	0.82
Nausea	11.23	2.78–45.35	0.00		1.25	0.48–3.24	0.65
Loss of appetite	2.14	0.61–7.54	0.24		1.05	0.42–2.60	0.92
Subjective pain	1.00	0.73–1.37	0.99		0.90	0.71–1.15	0.41
Nutritional status variables							
BMI (kg/m^2^)	0.74	0.61–0.90	0.00		0.69	0.58–0.82	0.00
Patients’ perception on weight status							
Underweight (reference)	1.00	–	–		1.00	–	–
Normal weight	0.19	0.05–0.66	0.01		0.08	0.01–0.62	0.02
Overweight	0.04	0.00–0.77	0.03		0.02	0.00–0.22	0.00
Self-perceived undernutrition	23.63	4.06–137.50	0.00		5.43	0.63–46.44	0.12
Weight loss 6 months (%)	1.19	1.07–1.32	0.00		1.13	1.02–1.25	0.02
FFMI (kg/m2)^d^	0.71	0.52–0.96	0.03		0.74	0.60–0.92	0.01
Undernutrition (yes vs no)	6.3	1.59–24.71	0.01		5.26	1.90–14.61	0.00
Functional variables							
Barthel score	1.07	0.92–1.26	0.38		0.93	0.83–1.05	0.26
Total dependence (reference)	1.00	–	–		1.00	–	–
Severe dependence	0.38	0.03–5.27	0.47		0.26	0.03–2.60	0.25
Moderate dependence	1.33	0.13–13.53	0.81		0.28	0.03–2.65	0.27
Slight dependence	0.62	0.04–8.70	0.72		0.23	0.02–2.46	0.22
FAC 0 (reference)	1.00	–	–		1.00	–	–
FAC 1	0.47	0.02–13.10	0.65		0.30	0.04–2.52	0.27
FAC 2	1.18	0.09–14.69	0.90		0.31	0.05–1.82	0.19
FAC 3	3.89	0.42–36.29	0.23		0.33	0.06–1.88	0.21
FAC 4	3.08	0.30-31.98	0.35		0.20	0.03–1.20	0.08
Feeding item: unable (reference)	1.00	–	–		1.00	–	–
Feeding item: needs help	0.16	0.01–3.26	0.23		2.67	0.14–49.76	0.51
Feeding item: independent	0.22	0.01–3.86	0.30		1.20	0.07–20.18	0.90
Difficulties with chewing or swallowing	3.96	1.06–14.82	0.04		2.61	0.66–10.37	0.17
Handgrip strength (kg)^f^	1.01	0.93–1.09	0.85		0.99	0.93–1.05	0.67
Psychological variable							
SF12 poor mental quality of life, MCS score < 50^g^	1.39	0.34–5.58	0.65		1.55	0.55–4.32	0.41
Measured on day 14							
Dietetic treatment variables							
Dietetic treatment	4.55	1.16–17.86	0.03		4.26	1.63–11.14	0.00
Sip/tube feeding	7.74	1.91–28.48	0.00		2.29	0.57–9.22	0.24

^a^Adequate protein and energy defined as: ≥ 1.2 g of protein/kg bodyweight per day (bodyweight was adjusted to a BMI of 27.5 for obese patients) and ≥ 85% of REE of Harris and Benedict + additional factor of 30%

^b^Adequate protein (per current guideline) and energy: ≥ 0.8 g of protein/kg bodyweight per day (bodyweight was adjusted to a BMI of 27.5 for obese patients) and ≥ 85% of REE of Harris and Benedict + additional factor of 30%

^c^Data missing for 1 patient

^d^Data missing for 16 patients

^e^Data missing for 2 patients

^f^Data missing for 5 patients

^g^Data missing for 3 patients

*FFMI* fat free mass index, *FAC* functional ambulation categories, *MCS score* mental component summary

Higher BMI, higher FFMI and patients’ perception on weight status (perceived normal weight or perceived overweight) were negatively associated with achieving adequate protein and energy intake. The statistical significant associations for adequate protein and energy intake are mostly in accordance with the results for achieving an intake of the current guideline (based on 0.8 g/kg bodyweight per day), with the exemption of the associations for elective orthopaedics, nausea, self-perceived undernutrition, difficulties with swallowing and chewing and poor mental quality of life (Table 2).

As to the concern for multicollinearity, MUAC was removed from the multiple logistic regression model given its less desirable VIF value and high correlation with BMI (VIF = 5.45, tolerance 0.18).

Due to missing data for some potential predictors, 74 patients were included in the multiple logistic regression analyses. The final model was statistically significant (chi square = 27.818, *P* < 0.000 with *df* = 3), explained 49% (Nagelkerke *R* ^*2*^) of the variance in adequate protein and energy intake, and correctly classified 88.6% of patients with an adequate protein and energy intake. Positive predictors included nausea and using sip or tube feeding, a negative predictor was having a higher BMI. Having a higher BMI was a negative predictor for achieving adequate protein (per current guideline) and energy intake (Table 3).

**Table 3:** Predictors for adequate protein and energy intake and for current guideline on day 14 in older rehabilitation patients

Adequate protein and energy	OR	95% CI	P
BMI (kg/m^2^)	0.677	0.525–0.873	0.003
Nausea	8.590	1.419–52.010	0.019
Sip/tube feeding	7.725	1.350–44.210	0.022
Sensitivity analysis: current guideline			
BMI (kg/m^2^)	0.689	0.582–0.815	0.000

Adequate protein and energy defined as: ≥ 1.2 g of protein/kg bodyweight per day (bodyweight was adjusted to a BMI of 27.5 for obese patients) and ≥ 85% of REE of Harris and Benedict + additional factor of 30%. Adequate protein (per current guideline) and energy: ≥ 0.8 g of protein/kg bodyweight per day (bodyweight was adjusted to a BMI of 27.5 for obese patients) and ≥ 85% of REE of Harris and Benedict + additional factor of 30%

When looking at predictors for adequate energy and adequate protein separately, a higher BMI was found to be the only significant (negative) predictor for adequate energy intake only (BMI OR = 0.672 *P* < 0.000, nausea OR = 4.496. *P* = 0.142 sip/tube OR = 4.287 *P* = 0.335). Predictors for achieving adequate protein only were the same as for both adequate protein and energy intake. For the 67 patients who did not have sip/tube feeding, BMI was the only (negative) significant predictor: OR = 0.661 *P* = 0.005.

In the sensitivity analysis, with the 11 patients discharged to home or self-care categorised as having adequate protein and energy intake; self-perceived undernutrition was the only positive predictor (OR = 13.2, *P* < 0.002). The variables sip/tube feeding and dietetic treatment were not included in the sensitivity model because these variables were not measured in the discharged patients.

## Discussion

This study investigated the protein and energy intake and predictors of an adequate protein and energy intake in older patients newly admitted to rehabilitation wards in nursing homes. Only 18% had an adequate protein and energy intake on the 14th day following admission. Fewer patients achieved an adequate protein intake than an adequate energy intake. Feeling nauseous during the first week of admission was associated with an increased likelihood of adequate protein and energy intake. Patients using sip or tube feeding were also more likely to have an adequate protein and energy intake than patients not using sip or tube feeding. A higher BMI was associated with a lower likelihood of adequate protein and energy intake.

Currently, the recommended daily intake of protein for older persons is 0.8 g/kg bodyweight [32, 33]. However, it has recently been suggested that older adults need more protein. The PROT-AGE study group recommends a higher protein intake (1.2 g/kg bodyweight per day) for older adults who are physically active [15] (as is the case in active rehabilitation). Similar to the findings of Dupertuis et al. [11] and Leistra et al. [12], our study suggests that this protein requirement is difficult to achieve for the older patients and more difficult to achieve compared to the energy requirement. Even if we had used the criterion of 0.8 or 1.0 g/kg bodyweight per day in our study, the percentage of patients meeting the protein and energy requirements would still be low; 59 and 46%. These data suggests that more attention is needed to increase the protein intake of older rehabilitation patients.

Patients with nausea in the first week following admission were more likely to have an adequate intake on day 14. This is opposite to the results of the study by Leistra et al. among hospital patients [12]. Their study measured patients’ sensation of feeling nauseous at about the same time as dietary intake was assessed (day 4 after hospital admission). Our study measured patients’ intake 14 days following admission. It is possible that patients suffering from nausea during the first week of admission will ‘catch-up’ on food intake in the following days. Another explanation might be that more attention is being paid to the dietary intake of patients feeling nauseous so that they are more likely to achieve their nutritional needs in the following days.

Similar to other studies [15], the use of sip or tube feeding on day 14 in was a positive predictor for adequate protein and energy intake. This finding would suggest a nutritional benefit of using of sip or tube feeding. However, considering the rather small number of patients receiving sip or tube feeding in our study and half of those patients had adequate nutrition on day 14, this finding should first be confirmed in larger studies.

Even though bodyweight was adjusted to a BMI of 27.5 kg/m^2^ when estimating the energy needs for those with a BMI > 27.5 kg/m^2^, having a higher BMI (and thus a higher absolute protein requirement), decreased the likelihood of adequate protein and energy intake.

A remarkable finding was that receiving dietetic treatment in the 2 weeks following admission did not increase the likelihood for adequate protein and energy intake on day 14. Dietetic treatment was significantly, positively related in the univariate analyses, but did not reach statistical significance in the multiple model. However, all patients receiving sip or tube feeding were also under dietetic treatment, and this specific treatment positively predicted adequate protein and energy intake. In addition, in patients with dietetic treatment but not receiving sip of tube feeding, treatment focused on other aspects than adequate protein and energy intake, such as diabetes mellitus, lactose intolerance and dietary fibre.

The strength of this study is that it provides unique data on the adequacy of nutritional intake of older patients in rehabilitation wards of nursing homes. A second strength is the prospective data collection and the numerous variables that could be included to examine their ability to predict adequate protein and energy intake. Although many variables were taken into account, we may have missed other potential predictors, including palatability of the menus and organizational factors, including skipped meals due to physiotherapy sessions and provided help with eating [34]. Another limitation is the small number of participants, resulting in less precise estimates which should be carefully interpreted.

## Conclusion

Only 18% of older nursing home rehabilitation patients had an adequate protein and energy intake on the 14th day following admission. Adequate protein intake was more difficult to achieve compared to adequate energy intake. Patients with higher BMI were less likely, while those using sip/tube feeding or experiencing nausea in the first week after admission were more likely to achieve an adequate protein and energy intake.

There is a need for further research to increase our understanding of the factors that contribute to an inadequate nutritional intake in this vulnerable older population. This information is needed to develop intervention strategies to improve dietary intake and reduce undernutrition in nursing home rehabilitation patients, thereby contributing to optimal rehabilitation outcomes.
